# Biological Mechanism on SIRT1/NLRP3/IL-18 Signaling Pathway of Acupuncture for Treatment of Ischemic Stroke with Center Poststroke Pain

**DOI:** 10.1155/2022/8958742

**Published:** 2022-08-26

**Authors:** Dan Zhou, Lanfang Zhang, Liwei Mao, Jingyu Cao, Jiaqiang Gao

**Affiliations:** ^1^Clinical Medical College of Acupuncture and Rehabilitation, Guangzhou University of Chinese Medicine, Guangzhou, China; ^2^Department of Rehabilitation, Guangzhou Tianhe District Chinese Medicine Hospital, Guangzhou, Guangdong 510665, China; ^3^Physiotherapy Department, Guangdong Hospital of Traditional Chinese Medicine, Guangzhou 510120, Guangdong, China

## Abstract

**Objective:**

To evaluate the effect of acupuncture on an animal model of ischemic stroke with central poststroke pain (CPSP) through Sirtuin 1 (SIRT1)/NOD-like receptor thermal protein domain associated protein 3 (NLRP3)/interleukin-18 (IL-18) signaling pathway.

**Methods:**

Data mining was performed with *R* package “edgeR,” “limma,” “pathview,” etc., from NCBI Gene Expression Omnibus (GEO) database. Sprague Dawley (SD) rats were divided into 4 groups: sham operation group (Sham group, *n* = 5), poststroke central pain group (CPSP group, *n* = 5), poststroke central pain + acupuncture group (AP group, *n* = 5), central pain after stroke + acupuncture + SIRT1 inhibitor EX527 group (EX527 group, *n* = 5). Pain behavior testing was performed to determine the mechanical withdrawal threshold (MWT). Quantitative real-time PCR (qRT-PCR) was performed to verify the data mining results from the GEO database.

**Results:**

The KEGG key pathway map was created using the *R* package “pathview” package, demonstrating that the expression levels of NLRP3's downstream inflammatory factors IL-18 were downregulated in both of siSIRT1 group compared to the control group and the NLRP3 reconstituted group compared to NLRP3 KO group. QRT-PCR results on animal models of CPSP ischemic stroke showed that the expression levels of SIRT1 were downregulated, the activation of the NLRP3 inflammasome was upregulated, and the expression levels of IL-18 were upregulated in the brain tissues of the surrounding area of the injury. As the pain threshold of CPSP rats was increased, the expression level of S1RT1 was upregulated, and the activation of NLRP3 inflammasome was downregulated. The expression level of IL-18 was downregulated after acupuncture treatment.

**Conclusion:**

Acupuncture may inhibit CPSP in an animal model of ischemic stroke by upregulating SIRT1 expression levels, inhibition of the activation of the inflammasome, and downregulating IL-18 expression levels.

## 1. Introduction

Stroke is an acute cerebrovascular disease characterized by high morbidity, complications, and high mortality. Central poststroke pain (CPSP), defined as pain associated with central nervous system damage, in general, is now preferred to describe the neuropathic pain after stroke [1]. CPSP is a neuropathic pain syndrome that can occur after a cerebrovascular accident. Most are caused by the sudden rupture of blood vessels in the brain or the blockage of local blood vessels in the body due to various factors and the inability of blood to flow into the brain. This makes the patient's language, hearing, and limb functions have certain obstacles, and the possibility of hemiplegia is high, which seriously affects the patient's daily living ability and usually occurs 6 months after ischemic stroke [2]. This neuropathic pain syndrome is characterized by pain and sensory abnormalities in the body parts that correspond to the brain territory that has been injured by the cerebrovascular lesion [3]. CPSP is one of the most common neurological complications after stroke, which accounts for 1% to 14% of stroke patients [4], and seriously affects the prognosis and quality of life of partitions. The most common presentations of CPSP are severe shoulder and hand pain. Shoulder and hand pain are common conditions among people who had a stroke, with its reported prevalence ranging from 12% to 49% [5, 6].

The pathogenesis of CPSP is not fully understood, and the treatment of CPSP is still based on drug therapy. Still, drug therapy can only partially relieve pain symptoms, and long-term use often leads to various adverse reactions. Acupuncture is a characteristic treatment of traditional Chinese medicine, which has the effect of channeling meridians and regulating qi and blood [7]. Acupuncture mainly stimulates the main acupuncture points on the affected limb, which plays a role in dredging the meridians, improving the joints, and reconciling qi and blood. At the same time, acupuncture and moxibustion are used together, focusing on regulating the yin and yang qi and blood of the liver, spleen, and kidney, dredging the meridians of the limbs, and achieving the purpose of both symptoms and root causes, which has been widely used in the rehabilitation treatment of common diseases in clinical departments [8].

Current clinical studies have confirmed that compared with drugs, acupuncture has a better therapeutic effect on CPSP and has a long-lasting effect and a low incidence of long-term adverse reactions [9]. However, the molecular mechanism of acupuncture for treating CPSP has not yet been elucidated, which has affected the further promotion of acupuncture. These patients suffer from allodynia and hyperalgesia after stroke, which is among the most troublesome sequelae of stroke [10]. Therefore, exploring the mechanism of action of acupuncture can lay a solid theoretical foundation for the prevention and treatment of CPSP by acupuncture, which is conducive to promoting the clinical application of acupuncture and has important clinical significance.

## 2. Method

### 2.1. Data Downloads from GEO

The RNA-seq data and annotation file were retrieved from NCBI Gene Expression Omnibus (GEO) (http://www.ncbi.nlm.nih.gov/geo/) with the *R* package “GEOquery.” RNA-Seq analysis of SIRT1 knockdown (KO) in the database of GSE171118 was performed based on the platforms of GPL23227 BGISEQ-500 (Homo sapiens) [11]. RNA-seq analysis of NOD-like receptor thermal protein domain associated protein 3 (NLRP3) KO and NLRP3 reconstituted in the database of GSE180707 was performed based on the platforms of GPL16791 Illumina HiSeq 2500 (Homo sapiens) [10].

### 2.2. Differentially Expressed Genes

The DEGs between the sirtuin 1 (SIRT1) group and control group of GSE180707 and between the NLRP3 KO group and NLRP3 reconstituted group of GSE171118 were screened and filtered out based on the *R* package “edgeR” [11] and “limma” [12, 13], requiring a fold-change (FC) > 1.0 or logFC <−1.0, and requiring the adjusted *P* values or false discovery ratio (FDR) < 0.05. The DEGs that differed - logFC >1 or < -1 and adjusted *P* values or FDR <0.05 - between the siSIRT1 group and control group were also identified using a volcano plot with top DEGs marked by gene symbols.

### 2.3. Pathview Analysis of Key KEGG Pathway

A selected critical pathway of interest, the NLRP3 inflammasome signaling pathway (https://www.kegg.jp/pathway/hsa04621+N00742) for the DEGs between the siSIRT1 group and control group of GSE180707, and between NLRP3 KO group, and NLRP3 reconstituted group of GSE171118, was also performed for visualization by *R* package “pathview” on Kyoto Encyclopedia of Genes and Genomes graphs. The statistical cutoff was set to a q value ≤ 0.05 [14, 15].

### 2.4. Experimental Animals

Sixty male healthy SPF-grade SD rats, weighing 180 to 220 g, were purchased from Junke Biology, Nanjing China, license number: SYXK (Su) 2019–0064. The rats were kept in a suitable environment for 7 days, maintaining indoor humidity at 50–60%, the temperature at 20–25 °C, alternating day and night for 12 h/12h. They were allowed to eat and drink freely.

### 2.5. Experiment Grouping

According to the random number table, SD rats (*n* = 20) were divided into 4 groups: sham operation group (Sham group, *n* = 5), poststroke central pain group (CPSP group, *n* = 5), poststroke central pain + acupuncture group (AP group, *n* = 5), central pain after stroke + acupuncture + EX527 group (EX527 group, *n* = 5).EX527 (6-chloro-2,3,4,9-tetrahydro-1H-carbazole-1-carboxamide) was one of the inhibitors of the Sirtuin family [16]. We selected Neiguan, Renzhong, and Sanyinjiao points and gave acupuncture stimulation 30 min/time, once a day for 5 days. The EX527 group was intraperitoneally injected with EX-5275 mg/kg 30 minutes before the daily acupuncture treatment, and the rest of the operations were the same as those in the EA group. Rats in the Sham and CPSP groups were not treated with acupuncture [17].

### 2.6. Establishment of Rat CPSP Model

We established a rat CPSP model with the literature by Shih et al. [18]. The rats in the CPSP group, EA group, and EX527 group were fixed on the brain stereotaxic device after intraperitoneal injection of 10% chloral hydrate. The hair on the top of the rat's head was shaved, and the skin was disinfected with 75% alcohol and 2% iodine. The first skin was cut along the sagittal suture, the subcutaneous tissue was carefully separated, and the periosteum was separated to expose the sagittal suture and bregma. Using the bregma as the origin, refer to the positioning coordinates (right thalamic ventro-posterior lateral nucleus (VPL): 3.5 mm behind the bregma, 3.2 mm next to the midline, and 6.2 mm below the anterior bone). Under the stereotaxic guidance, the VPL was injected with type IV collagenase (dissolved 0.125U type IV collagenase in 1 *μ*l saline), then the wound was cleaned, and the scalp was sutured. Rats in the Sham group were injected with 1*μ l* sterile saline.

### 2.7. Methods of Acupuncture Treatment

The rats were restrained and fixed on a self-made fixing device; insert the acupuncture needles into the Neiguan, Renzhong, and Sanyinjiao points of the rats in sequence. Neiguan acupoint was located on the inner side of the rat's forelimb, between the ulna fusiform suture about 3 mm away from the wrist joint, and there was one acupuncture point on the left and right sides. Renzhong acupoint was located about 1 mm in the middle of the rat's cleft lip and nose tip. Sanyinjiao was located about 1 cm above the tip of the medial malleolus of the rat's hind limbs, and there was an acupuncture point on the left and right sides. Each treatment was performed for 30 minutes.

### 2.8. Pain Behavior Testing with the Determination of Mechanical Withdrawal Threshold (MWT)

The rats of each group were placed in a mesh cage with a metal mesh at the bottom at 1d before modeling and 1d, 3d, and 5d after modeling. After adapting to the environment for 30 minutes, we used an electronic mechanical pain meter to slowly and vertically puncture the center of the left hind foot of the rats. If the rats had the foot withdrawal reaction, it was positive, and the mechanical stimulation threshold was recorded. We repeated the operation at an interval of more than 10 minutes. The average value was taken as the mechanical withdrawal threshold of the rats until 3 positive reactions were recorded.

### 2.9. RNA Extraction and Quantitative Real-Time PCR

Quantitative real-time PCR (qRT-PCR) was performed to verify the results of data mining of the GEO database. After the pain behavior measurement was completed, the rats were anesthetized by intraperitoneal injection of 10% chloral hydrate. The skin was cut quickly and carefully to avoid pressing the brain tissue after the rat's neck was cut off with a decapitator. Then, we insert the curved forceps into the gap between the skull and the medulla oblongata, lift off the rat's skull, and remove the remaining skulls. After full exposure of the rat brain tissue, peeling is performed, then the brain tissue is removed, the filter paper absorbs the water and blood on the surface, and it is placed in a freezer at -80°C for storage. The manufacturer's instructions extracted total RNA from brain tissues using Trizol reagent (Takara, Kyoto, Japan). cDNA was synthesized using the PrimeScript reverse transcription-polymerase chain reaction kit (Takara, Kyoto, Japan). The qRT-PCR was performed in SYBR Green Master Mix (Roche). The 2−∆∆Ct method was performed to analyze the data, and GAPDH was used as a loading control. The primer sequences are listed in Table 1.

## 3. Statistical Analysis

All values were expressed as mean ± SEM. The statistical significance was determined by Student's *t*-test, performed by *R* package “ggpubr” and “ggplot2” with the function of stat_compare_means. *p* < 0.05 was taken as statistically significant.

## 4. Results

### 4.1. Identification of DEGs Related to SIRT1

**Figure 1:**
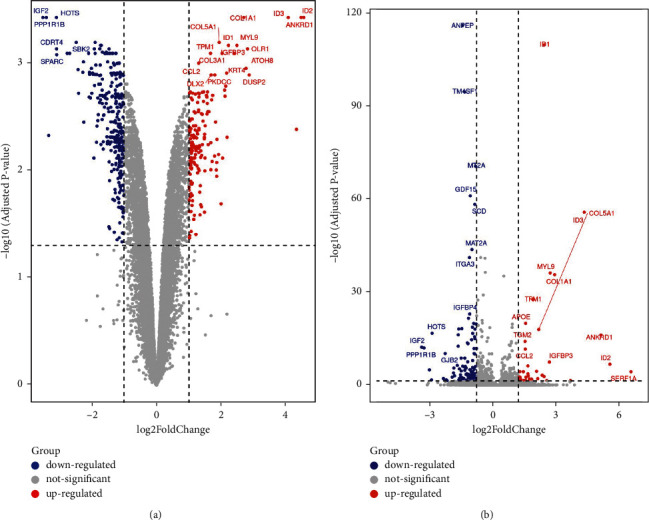
Volcano plot shows the DEGs between siSIRT1 and normal control samples. Fold change (FC) > 1 (in red) or < −1 (in blue) with adjusted *P* values or FDR <0.05 are differentially increased or reduced in siSIRT1 samples relative to normal control samples. (a) Volcano plot performed by limma; (b) volcano plot performed by edgeR.

The gene expression dataset of GSE171118, which contains 2 siSIRT1 samples and 2 normal control samples, was analyzed in the limma package with threshold parameters defined as fold change (FC) > 1 or < -1 and the adjusted *P* values < 0.05. In total, 465 DEGs (173 high expression genes and 292 low expression genes) were identified between siSIRT1 samples and normal control samples, and there were 78 high expression genes and 170 low expression genes with the same threshold in the edgeR package (Table 2). Visualization was performed with volcano plot analysis to show the DEGs screened by limma (Figure 1) and edgeR (Figure 1(b)), respectively.

### 4.2. KEGG Pathway Analysis

From the path view KEGG pathway maps, it was observed that the expressions levels of NLRP3's downstream inflammatory factors interleukin-18 (IL-18) were downregulated in both of siSIRT1 group compared to the control group and NLRP3 reconstituted group compared to NLRP3 KO group, suggested that IL-18 expression levels were inhibited probably by SIRT1 expression activated and promoted. However, the expression levels of NLRP3 did not change significantly in the siSIRT1 group compared to the control group in the dataset of GSE171118.

### 4.3. Changes in Pain Behavior of Rats Measured by MWT

**Figure 2:**
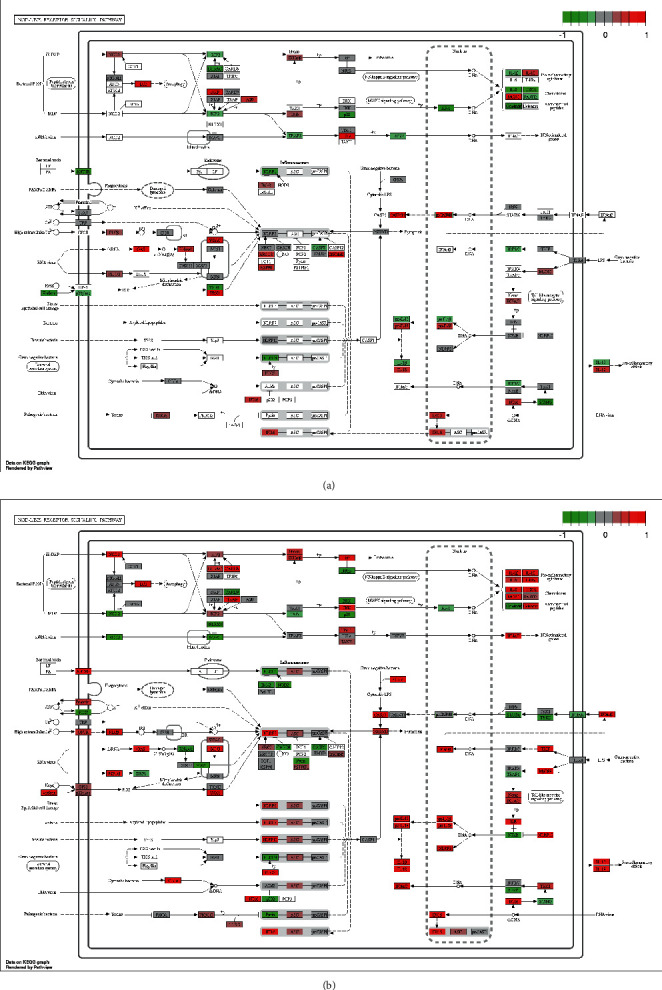
KEGG Pathview analysis of molecular signature of NLRP3 inflammasome signaling pathway, (a) Pathview analysis of siSIRT1 group compared to Control group; (b) Pathview analysis of NLRP3 reconstituted group compared to NLRP3 KO group; KEGG pathway analysis demonstrating upregulation or downregulation of multiple components of the complement pathway. Each box was one gene; the green boxes show the downregulated gene expression of mRNA while the red boxes show the upregulated gene expression.

**Figure 3:**
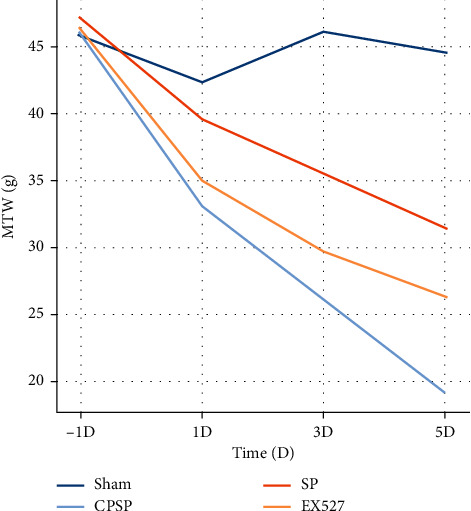
Changes in mechanical pain threshold in rats after establishing a central pain model after stroke. Compared with the Sham group, the MWT value of the left plantar in the CPSP group was decreased (*P* < 0.05); compared with the CPSP group, the MWT value in the AP group was increased (*P* < 0.05); compared with the AP group, MWT value in EX527 group was increased (*P* < 0.05).

In the experiment on pain behavior, MWT values of the rats in the Sham group, CPSP group, AP group, and EX527 group were measured at 1d before modeling and at 1d, 3d, and 5d after modeling separately. We found that there was no statistical difference in MWT value of the rats in each group before the establishment of CPSP model, and there was no significant difference in MWT value of rats in the Sham group before and after modeling. Meantime, MWT value of the rats in the CPSP, EA, and EX527 groups was significantly decreased at d1-d3 compared with the Sham group; MWT value of the rats in the AP group was significantly increased compared with the CPSP group, while Figure 2 MWT value of the rats in the EX527 group was significantly decreased compared with the AP group (Figure 3).

### 4.4. mRNA Levels of NLRP3, SIRT1, and IL-18

**Figure 4:**
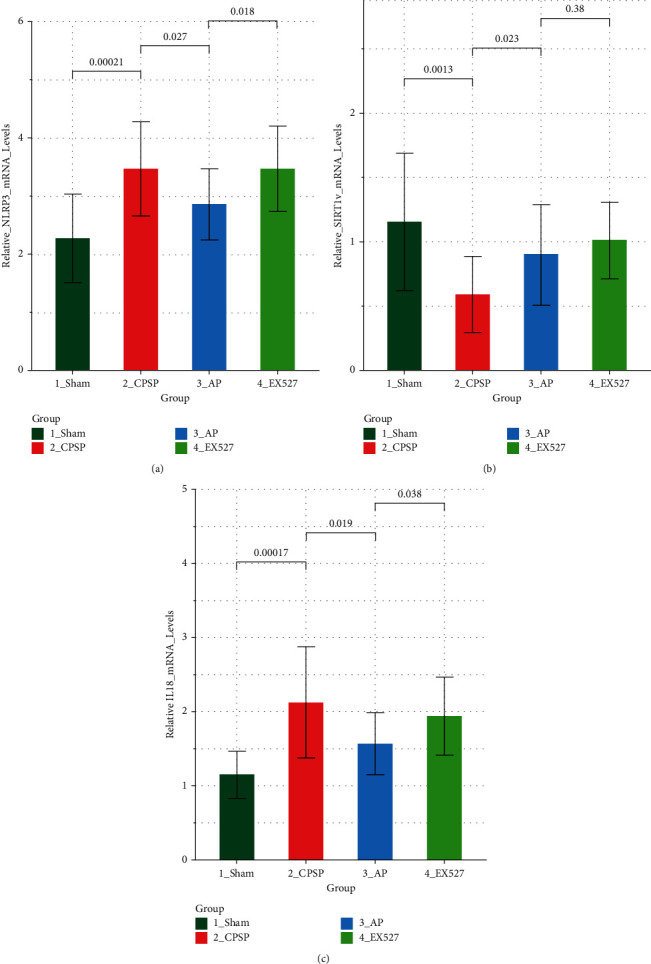
NLRP3, SIRT1 and IL-18 mRNA expression. (a) NLRP3, (b) SIRT1, and (c) IL-18 mRNA expression levels, which were normalized to GAPDH. *N* = 5 in each group. Studies were repeated three times with highly similar results.

The mRNA expression levels of NLRP3, SIRT1, and IL-18 on an animal model of ischemic stroke with CPSP were measured by qRT-PCR to validate the data mining findings from GEO. As illustrated in Figure 4(c), the expression levels of SIRT1 was downregulated and the activation of NLRP3 inflammasome was upregulated, the expression levels of IL-18 was upregulated in brain tissue around the injured area in animal model; as the pain threshold of CPSP rats was increased, the expression levels of S1RT1 were upregulated and the activation of NLRP3 inflammasome was downregulated, and the expression levels of IL-18 were downregulated after acupuncture treatment.

## 5. Discussion

The neuropathic pain syndrome is caused by the corresponding ischemic or hemorrhagic lesions affecting the spinothalamic pathway [19]. The pathogenesis is not clear. It is usually characterized by sensory disturbances, persistent or intermittent pain, hyperalgesia, and hypersensitivity [20]. Studies have found that IL-17 can be detected in different central nervous system inflammatory states and is involved in neuropathic pain associated with multiple cirrhoses and autoimmune encephalomyelitis [21]. IL-17A can induce ATG5 and ATG7 to promote inflammation. It can also directly cause endothelial cells to release chemokines, thereby increasing leukocyte migration and the permeability of the blood-brain barrier, causing brain injury damage [22]. Based on current studies, we believe that CPSP was closely related to inflammation in the central nervous system. However, due to the complexity of inflammation signaling pathways, the mechanism has not yet been fully elucidated before.

SIRT1, as a NAD-dependent protein deacetylase, is involved in inflammatory responses, oxidative stress, and other processes. It also plays a cerebroprotective role in various inflammatory diseases of the nervous system by inhibiting the activation of the NLRP3 inflammasome and the immune upstream regulator IL-18 of NLRP3. SIRT1 can mediate gene silencing and life extension in yeast and fruit flies [23]. Homologs, including SIRT1-7, can catalyze protein deacetylation or adenosine diphosphate ribosylation. Its characteristic is that sirtuins need oxidized nicotinamide adenine dinucleotide (NAD+) undergo an enzymatic reaction to generate nicotinamide (NAM) as a negative feedback inhibitor [24]. Because of this NAD + dependence, Sirtuins are classified as a group II group egg white deacetylase (Histon deacetylases, HDACs). SIRT1 is a kind of deacetylation that relies on NAD + enzymes, a member of the SIRT family, which can regulate oxidative respiration, inflammation and various physical environments, and then participate in the regulation of various activities of the body. It is activated in a NAD + dependent and non-NAD + dependent manner. In addition, SIRT1 can target and regulate various transcription factors, such as NF-kB, E2F1, p53, FOXOs, HIF-1, PGC-la, LXR, Myod [25]. Under environmental stress, SIRT1 is transcribed to protein stimulation. Every step of life is dynamically regulated. At the post-transcriptional level, the HuR gene (also known as embryo-like lethal abnormal vision gene, ELAVL1) stabilizes SIRT1 by interacting with its 30-UTR mRNA SIRTI. Its activity is also regulated in many ways, such as its phosphorylation [26] and ubiquitination [27], or with AROS's [28] interaction with DBC1 [29]. The final expression and function of SIRT1 depend on the regulation of the redox festival. The research suggests that the use of bone marrow cell-specific SIRT1 knockout mouse models can be used in macrophage ablation. SIRT1 presents NF-KB hyperacetylation, which leads to increased transcriptional activation of proinflammatory target genes [30]. The results of this study by establishing a model of CPSP in rats and performing interventional drug therapy in different groups are consistent with our speculations on reducing the pain threshold in CPSP rats. The expression level of SIRT1 in brain tissue was downregulated, and NLRP3 and IL-18 expression levels were upregulated. Acupuncture could increase the pain threshold of CPSP rats, promote the expression levels of SIRT1, reduce the expression levels of NLRP3 and IL-18, and reduce the pain symptoms of rats. We found that the therapeutic effect of acupuncture could be reversed by a SIRT1 inhibitor, indicating that SIRT1 may play an important role in the process of acupuncture treatment of CPSP.

NLRP3 inflammasome is a polyprotein complex with NLRP3, ASC, and procaspase-1 as the core protein. When activated, it can rapidly induce the maturation and secretion of cytokines to trigger the body's inflammatory response [31]. It was found that the mouse lung tissue showed obvious pathological damage. At the same time, the expression levels of NLRP3 inflammasome and IL-18 in the lung tissue were upregulated by using intratracheal infusion of lipopolysaccharide to induce a mouse model of acute lung injury. The degree of lung injury in mice was significantly reduced after treatment with the NLRP3 inflammasome inhibitor MCC950. At the same time, the activation of NLRP3 inflammasomes in the lung tissues was reduced, and the expression levels of IL-18 were downregulated, indicating that the activation of NLRP3 inflammasomes was inhibited, which play a protective role to reduce the subsequent secretion of IL-18, and reduce the levels of inflammation in the body [32].

In another animal experiment, the middle cerebral artery occlusion method was performed to establish a mouse cerebral ischemia-reperfusion model, and it was found that the expression of low-density lipoprotein receptor (LDLR) in the brain tissue of the model mouse was downregulated and induced the activation of NLRP3 inflammasome and the subsequent maturation, and the release of IL-18 eventually led to neuronal apoptosis. After treatment with the NLRP3 inflammasome inhibitor CY-09, the expression levels of IL-18 in the brain tissue were decreased, and the number of neuronal apoptosis was decreased, indicating that the NLRP3 inflammasome in the brain tissue can also regulate the maturation and secretion of IL-18 and other cytokines, and then participate in the body's inflammatory response. IL-18 are common proinflammatory cytokines that are closely related to a variety of neuropathic pain [33].

The present study observed that the pain threshold of CPSP rats increased after acupuncture treatment, the expression levels of SIRT1 in the brain tissue was upregulated, and the expression levels of NLRP3 and IL-18 were downregulated significantly. The pain threshold of rats was reduced, the expression levels of SIRT1 in the brain tissue were downregulated, and the expression levels of NLRP3 and IL-18 were upregulated.

There are also some shortcomings in this study. Although this study found that the improvement of stroke by acupuncture was related to the upregulation of SIRT1 expression levels in brain tissue and the significant downregulation of the expression levels of NLRP3 and IL-18, the specific in-depth mechanisms were not further studied. In addition, acupuncture effects may vary from acupuncture point to acupuncture point, and this study did not investigate. These will be the problems that need to be further solved in future research.

## 6. Conclusion

Acupuncture treatment of CPSP could inhibit the activation of the inflammatory body of NLRP3, and NLRP3's downstream inflammatory factors of IL-18 expression were inhibited.

## Figures and Tables

**Table 1:** Primer sequences used for the quantitative real-time polymerase chain reaction.

Gene	Forward primer (5′-3′)	Reverse primer (5′-3′)
SIRT1	GCTCGCCTTGCTGTGGACTTC	GTGACACAGAGATGGCTGGAACTG
NLRP3	CCAGGAGTTCTTTGCGGCTA	GCCTTTTTCGAACTTGCCGT
IL-18	CAACCGCAGTAATACGGAGC	GATTCGTTGGCTGTTCGGTC
GAPDH	ACAGCAACAGGGTGGTGGAC	TTTGAGGGTGCAGCGAACTT

**Table 2:** Results of differentially expressed mRNA of GSE171118.

Compare	Style	Identification tools	Log2FC_Cutoff	adjusted_Pvalue(FDR)_Cutoff	All differential expressed number	Upregulated number	Downregulated number	Total number after merging
siSIRT1 group versus normal control group	mRNA	limma	>1 or < −1	0.05	465	173	292	466
		edgeR	>1 or < −1	0.05	132	37	95	

## Data Availability

The datasets used and analyzed during the current study are available from the corresponding author upon reasonable request.
